# Genetic identity and genotype × genotype interactions between symbionts outweigh species level effects in an insect microbiome

**DOI:** 10.1038/s41396-021-00943-9

**Published:** 2021-03-12

**Authors:** Melanie R. Smee, Sally A. Raines, Julia Ferrari

**Affiliations:** 1 Department of Biology, University of York, York, UK; 2 Present Address: Microbiology Department, Cornell University, Ithaca, NY USA

**Keywords:** Microbial ecology, Community ecology, Symbiosis, Microbiome, Evolution

## Abstract

Microbial symbionts often alter the phenotype of their host. Benefits and costs to hosts depend on many factors, including host genotype, symbiont species and genotype, and environmental conditions. Here, we present a study demonstrating genotype-by-genotype (G×G) interactions between multiple species of endosymbionts harboured by an insect, and the first to quantify the relative importance of G×G interactions compared with species interactions in such systems. In the most extensive study to date, we microinjected all possible combinations of five *Hamiltonella defensa* and five *Fukatsuia symbiotica* (X-type; PAXS) isolates into the pea aphid, *Acyrthosiphon pisum*. We applied several ecological challenges: a parasitoid wasp, a fungal pathogen, heat shock, and performance on different host plants. Surprisingly, genetic identity and genotype × genotype interactions explained far more of the phenotypic variation (on average 22% and 31% respectively) than species identity or species interactions (on average 12% and 0.4%, respectively). We determined the costs and benefits associated with co-infection, and how these compared to corresponding single infections. All phenotypes were highly reliant on individual isolates or interactions between isolates of the co-infecting partners. Our findings highlight the importance of exploring the eco-evolutionary consequences of these highly specific interactions in communities of co-inherited species.

## Introduction

Interactions between eukaryotes and microbes are as ubiquitous as they are varied [1, 2]. Many mutualistic microbes provide essential benefits to their host, often resulting in obligate symbiotic associations [3, 4]. Conversely, there is a wide array of parasitic or pathogenic interactions between host and microbe [5–7], but intermediary situations are perhaps most common, whereby a “facultative” symbiont may provide conditional benefits to a host, but may also incur a cost [8]. In recent decades, research has uncovered many fascinating roles of microbes, yet often such research is conducted on a tractable one host—one microbe system. It is widely recognised now that this is often not reflective of the true dynamics in a natural system, where multiple microbes interact inside, on, or indirectly with a host and have knock-on effects at the community level [9].

Most microbiomes are complex and species rich [2, 10] but inevitably research on these microbial communities is usually descriptive and correlational and thus hard to interpret for specific interactions e.g. [6, 11]. We currently have a limited understanding of microbe-microbe interactions within hosts, and microbe-host interactions when there are multiple microbes present [12–14]. Each bacterial symbiont may be characterised as beneficial individually, but when co-existing with another may become surplus to needs [15–17] or even costly to the host in some environmental situations [18]. Like all ecological communities, microbiomes not only consist of multiple species, but each species is typically composed of several genotypes [19, 20]. A recent meta-analysis across biological systems showed that intraspecific variation can affect ecological processes as much as the presence of a particular species, but also highlighted that the number of data sets that allow such analyses is limited and biased towards a small number of systems [21]. The relative importance of genotype × genotype (G×G) compared to that of species interactions is even less well understood. G×G interactions have the potential to lead to more complex food webs [22] and hence altering ecological processes, which likely leads to eco-evolutionary feedback [23]. This is probably particularly important when the interacting species are closely linked and frequently co-inherited such as in host-associated microbiomes [24] or communities associated with long-lived plant species [25].

Systems of moderate complexity are needed in order to gain an understanding about specific interactions and processes within a host. Arthropods are an ideal study system as they often harbour multiple facultative symbionts [26–28]. In particular, the pea aphid (*Acyrthosiphon pisum*) provides a model system as phenotypes conferred by single infections of facultative symbionts are well-characterised and resident microbes are few [29]. Aside from the obligate symbiont, *Buchnera aphidicola*, which provides essential amino acids to the host [30, 31], there are at least seven known facultative symbionts [32]. An individual host typically harbours 1-4 facultative symbiont species [33]. These provide a variety of benefits such as protection from parasitoid wasps [34] or fungal pathogens [35], and recovery after exposure to high temperatures [36]. Co-infections are common and dynamic, and can be gained and lost over the course of a season [33]. In addition, different environmental conditions may favour certain combinations of symbionts [37], and in general some associations are more common than expected by chance [38].

The pea aphid’s consortium of symbionts includes two *Gammaproteobacteria*: *Candidatus* Hamiltonella defensa (previously T-type or PABS, henceforth *Hamiltonella*) [39], and *Candidatus* Fukatsuia symbiotica (X-type or PAXS, henceforth *Fukatsuia*) [40]. *Fukatsuia* frequently occurs in co-infections with *Hamiltonella* [16, 41], and the two have different effects on hosts. *Hamiltonella* is known for its protection against the parasitoid wasp *Aphidius ervi* through the implementation of a phage (APSE) [34, 42–45]. *Fukatsuia* can provide several ecological benefits dependent on the isolate, from protection from natural enemies to recovery after exposure to high temperatures, but also imposes costs on the host [16, 46]. In US populations of the pea aphid, one isolate of *Fukatsuia* appears to hitchhike alongside *Hamiltonella* without providing any benefits [16]. This previous study was conducted with one genotype each of *Hamiltonella* and *Fukatsuia*, on one aphid host genotype. Even within single infections, genetic identity is known to play a large role in ecological outcomes, especially for strains of *Hamiltonella*. Depending on the APSE variant, strains of *Hamiltonella* display varying levels of protection against *A. ervi* [47, 48]. When further combined with different aphid genotypes possessing different levels of endogenous defences, the best defence strategy against *A. ervi* varies for each host genotype, with some performing better with no *Hamiltonella*/APSE at all [49]. Hence, the genetic identity of APSE, *Hamiltonella*, and the host all interplay to provide varying levels on which selection may act in the field.

Interactions among co-infecting symbionts may play a critical role in determining distributions in natural populations. Our knowledge on co-infections is limited: while there are a few studies that have attempted to unravel these interactions experimentally, a systematic investigation is lacking. Łukasik et al. [50] found that co-infection with *Hamiltonella* did not affect the protection from a fungal pathogen conferred by other symbionts, and McLean et al. [17] similarly demonstrated that in co-infections the phenotype was equal to that of the more protective symbiont. In contrast, *Hamiltonella* and *Serratia symbiotica* provided greater resistance to *A. ervi* when co-infecting, but this coincided with severe fecundity costs and higher symbiont densities competing within the host [18]. These interactions also become more complicated when considering different host genotypes [14]. Despite employing only one host genotype, the current study is the first to systematically address the importance of GxG interactions between co-infecting bacterial symbionts.

As explained above, it is clear that facultative symbionts rarely work alone, and that there is much ecologically relevant variation at the isolate level [51, 52]. Here, we investigate the interactions between multiple isolates of two different species of facultative bacterial symbiont within a single lineage of an insect host facing several ecological challenges: a parasitoid wasp, a fungal pathogen, heat shock and fecundity on different host plants. We tested (i) whether the phenotypic variation due to isolates and isolate specific interactions is comparable to that due to species and species interactions. We then tested the hypotheses that (ii) the costs and benefits conferred by a symbiont are altered when two symbionts coexist and that (iii) these alterations depend on the genotype of the coexisting species. Finally, (iv) we asked whether, in general, a beneficial symbiont can maintain its benefit for the host in the presence of a less beneficial symbiont, or whether its benefit is reduced. Similarly, we tested whether the presence of the least beneficial symbiont causes unrecoverable negative impacts on a host’s phenotype or whether this can be rescued by the more beneficial partner. We present these final analyses in Text S8 and discuss the evolutionary implications in the supplementary material.

## Materials and methods

### Creation and maintenance of aphid lines

Pea aphids reproduce parthenogenetically under spring and summer conditions and it is thus possible to keep essentially genetically identical lines in the lab. The aphid lines were kept in culture on seedlings of *Vicia faba*, a plant species that almost all pea aphids, *Acyrthosiphon pisum* (Harris), perform well on [53]. Conditions were kept at 15 °C and light:dark 16:8 h.

We created 36 aphid lines with all possible combinations of five isolates of *Hamiltonella* and five isolates of *Fukatsuia*, corresponding single infections and an uninfected line (Text S1; Table S1). A single pea aphid clonal line (218) was used as the host. This line was naturally infected with both *Hamiltonella* and *Fukatsuia* (Table S2). An antibiotic cocktail was used to cure the aphids of these natural symbionts [54] before establishing all experimental lines. For details of DNA extraction and PCR protocols see Text S2 and Table S3. It should be noted that one of the donor lines (217) was originally co-infected with *Fukatsuia* and *Spiroplasma*. The latter unfortunately also transferred during artificial infections and is consequently found in all lines containing this *Fukatsuia* isolate (*F*_5_). As we are unable to cure *Spiroplasma* from individuals, this is taken into account in the analysis by running all models with and without the lines harbouring *Spiroplasma* (Text S7). Almost all of the co-infected lines were stable in the laboratory, with the exception of two lines, which were lost after ~9–12 months and recreated before experiments continued (Text S6, Fig. S1).

### Susceptibility to the parasitoid Aphidius ervi

Groups of 30 three- to four-day-old aphids were exposed to individual *A. ervi* females as detailed in Heyworth and Ferrari [46] and Text S3. Ten days after exposure the number of “mummies” formed by parasitoid larvae developing inside successfully parasitised aphids were counted, as well as the number of surviving non-parasitised aphids. An average of 4.4 (range of 3–6) replicates were carried out for each aphid line.

### Susceptibility to the fungal pathogen Pandora neoaphidis

Aphids were subjected to spores of the fungal pathogen *Pandora neoaphidis* Humber (Zygomycetes; Entomophorales) (isolate reference X4, supplied by Jason Baverstock and the Rothamsted Research collection). As detailed in Heyworth and Ferrari [46] and Text S4, groups of 20 ten-day-old apterous aphids were exposed to sporulating cadavers for 90 min. After 10 days plants were checked regularly for infected and sporulating aphid cadavers, and after a total of two weeks the remaining aphids left alive were counted. This assay was repeated an average of 5.8 (range of 4–9) times for each aphid line.

### Fecundity on a “specialist” and a “generalist” host plant

All aphid lines used in the study were collected from *Medicago sativa*, the “specialist” host plant. Adult aphids were placed in Petri dishes containing leaves of *M. sativa* suspended in 2% agar and left to reproduce overnight. Groups of five offspring were subsequently kept on *M. sativa* leaves until final instar when a single young apterous adult was placed individually on leaves of *M. sativa*. Offspring were then counted every 2 or 3 days until two successive counts of one or zero offspring, at which point that individual was considered to have stopped reproducing. There were six replicates for each line. Fecundity on the “generalist” host, *V. faba*, was measured from the control treatment lines in the heat shock assays described below.

### Performance after heat shock

To assess symbiont effects on the survival and fecundity of aphids after heat shock, groups of ten two-day-old aphids from each of the 36 lines were subjected to a temperature regime following the protocol in Heyworth and Ferrari [46]. A replicate consisted of a group of ten aphids and was either kept as a control group at 20 °C or subjected to heat shock, whereby the temperature was raised consistently from 20 °C to 38.5 °C over a 2-h period, then remained at 38.5 °C for 4 h before being lowered back to 20 °C over another 2-h period. All aphids, including controls, were moved onto fresh plants on the following day. Seven days later the surviving aphids were counted and one apterous individual per cage was kept for fecundity counts as described above. Seven to eleven replicates (mean 8.3) were conducted for each aphid line and heat treatment.

### Data analysis

A series of analyses were conducted on each dataset (Table S4), using R version 3.4.3 and RStudio Version 1.1.383 [55]. Susceptibility to the parasitoid wasp *A. ervi* and the fungal pathogen *P. neoaphidis* were calculated as the proportion either mummified or sporulating, respectively, out of the original number of aphids included in each assay. Survival after heat shock was measured as the proportion of the ten aphids alive at 8 days post heat treatment, and hence at reproductive maturity. The final three datasets were all lifetime fecundity count data (on alfalfa; on broad bean; and after heat shock).

We ran an initial model to determine the proportion of deviance in the phenotypic data that was explained by each of the following factors: symbiont species, symbiont isolate, occurrence of a co-infection, or specific combination of infecting isolates. We used a generalized linear model (GLM), which included experimental block as a fixed factor as well as the presence of each symbiont and the isolate of each symbiont as well as interaction terms of symbiont presence and symbiont isolates. From a base model including only the effect of experimental block, we sequentially added terms and recorded the deviance explained by each and calculated their proportions of the explained total (not including any residual deviance in the model, or that explained by experimental block).

We then investigated whether infection status (i.e., single infection with either *Hamiltonella* or *Fukatsuia*, co-infected, or uninfected) was important. We used generalized linear mixed models (GLMMs) and the R package ‘lme4’ [56] with aphid line and experimental block as random factors and infection status as a fixed factor (Text S5.1). To determine if the specific isolate of each species of symbiont or the interaction between them was important, we again used GLMMs but with only experimental block as a random factor and isolate as a fixed factor. For each scenario we performed three analyses, two for the five isolates of either species, and one using just the co-infected lines; the latter including an interaction term of *H*_isolate_ × *F*_isolate_ (Text S5.2). See Table S4 for specific models for each dataset.

We ran all models with all available data, and also without the six aphid lines also harbouring *Spiroplasma* (i.e., lines with *F*_5_; Table S5; Text S7). In general, these analyses yielded very similar results, so we only present the analyses using the complete datasets in the main text. We also ran a simple linear mixed model to compare fecundity after heat shock with fecundity on control plants kept at 20 °C, to illustrate whether infection status may affect recovery when aphids are subjected to extremes of heat.

## Results

### The importance of genetic identity and genetic interactions

**Fig. 1:**
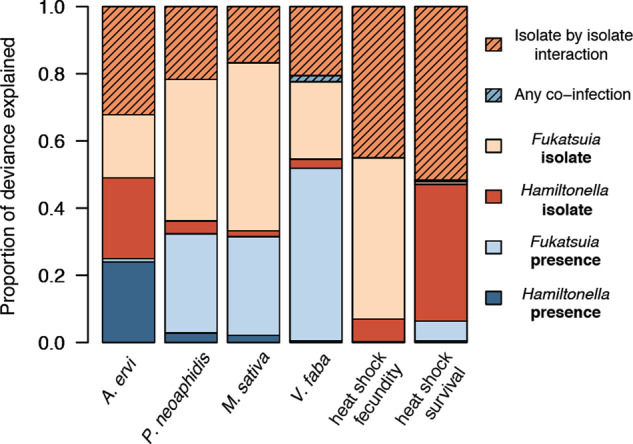
Model deviance explained by different symbiont statuses. The proportion of the deviance in generalised linear models (GLMs) for each experimental assay explained by just the presence of either of the two symbiont species, the individual isolates, the occurrence of any co-infection, and the co-infection between specific isolates.

For all the ecological scenarios, there was variation among isolates or interactions between isolates that contributed significantly to the aphid phenotype. For all traits, we first asked how much of the phenotypic deviance was explained by these isolate specific effects compared to species presence or interactions between species (Fig. 1). The presence of both species combined explained between 0.16 and 51.9% of the variation in a given trait, with *Hamiltonella* presence predicting resistance to *A. ervi*, whereas *Fukatsuia* predicted resistance to the fungal pathogen, and fecundity on both plants. In most cases, the contribution of isolate specific effects (explaining 25.8 to 54.7% of the deviance) was greater than species level effects with the exception of fecundity on *V. faba*. Interestingly, carrying a co-infection, irrespective of isolate, explained almost none of the deviance in any trait (a maximum of 1.8% for fecundity on *V. faba*). However, if the particular combination of isolates is taken into account, the interaction explained between 17 and 52% of the deviance in each dataset, although this component is only significant for the susceptibility to natural enemies and not for the other traits (Fig. 1).

### Susceptibility to the parasitoid A. ervi

**Fig. 2:**
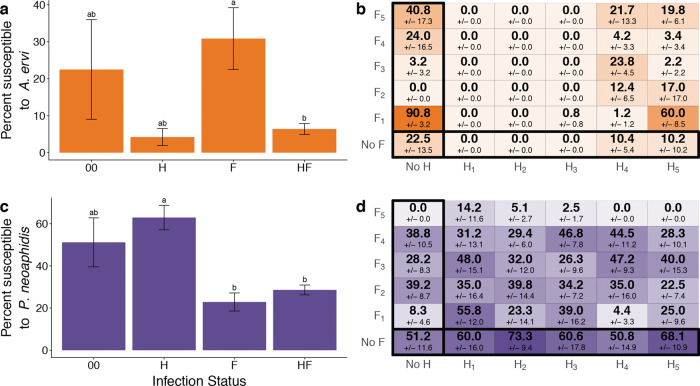
Susceptibility to natural enemies, by infection status and by individual aphid line (± standard error). **a-b** Mean percentage of aphids mummified when exposed to a female parasitoid wasp. An average of 4.4 (range of 3–6) replicates were carried out for each aphid line; (**c-d**) mean percentage of aphids susceptible to the fungal pathogen *Pandora neoaphidis*, taken as those forming cadavers after exposure to fungal spores. Repeated an average of 5.8 (range of 4–9) times for each aphid line. 00 = uninfected aphid line; H = *Hamiltonella defensa*; F = *Fukatsuia symbiotica*; HF = co-infection. For tables of individual lines, shade of colour is relative to value in cell (darker = more susceptible to natural enemy), note that while there is substantial variation between lines, for some lines there are also relatively large standard errors.

The infection status of aphid lines was important, as single infections of *Fukatsuia* tended to increase susceptibility and aphids carrying *Hamiltonella*, either in single or co-infections, benefitted from increased resistance (Fig. 2a; GLMM; *χ*^2^_3_ = 8.52, *p* = 0.03). In singly infected hosts, isolates of *Hamiltonella* varied in the level of resistance provided (Fig. 2b; GLMM: *χ*^2^_4_ = 18.89, *p* < 0.001), and all but *H*_*4*_ were significantly lower than the uninfected line. We found unexpectedly high variation in the level of protection conferred by single *Fukatsuia* isolates, which varied significantly from complete protection (isolate *F*_*2*_) to increased susceptibility for isolate *F*_*1*_ (Fig. 2b; GLMM: *χ*^2^_4_ = 251.85, *p* < 0.001).

For aphids co-infected with both species of symbiont, both the isolate of *Fukatsuia* (GLMM; *χ*^2^_4_ = 33.18, *p* < 0.001) and the isolate of *Hamiltonella* (GLMM; *χ*^2^_4_ = 186.01, *p* < 0.001) affected their likelihood of mummifying, as well as the interaction between the two (GLMM; *χ*^2^_16_ = 114.43, *p* < 0.001). This is clear when comparing across the isolates of *Hamiltonella*. Three of the five isolates showed 99–100% protection in both single and double infections, regardless of the co-infecting *Fukatsuia* isolate (Fig. 2b). However, in other combinations the presence of specific isolates of each species alter the susceptibility to *A. ervi*. For example, *H*_*5*_ is the only *Hamiltonella* isolate unable to fully reduce the increased susceptibility of aphids to *A. ervi* infected with *F*_*1*_. Yet when *H*_*5*_ co-infects with the fully protective *F*_*2*_ isolate susceptibility unexpectedly increases beyond that of both single partners.

### Susceptibility to the pathogen P. neoaphidis

The susceptibility to *P. neoaphidis* was affected by the infection status of the aphid: aphids carrying *Fukatsuia* on its own or in a co-infection were on average more resistant to the fungal pathogen than those that harboured only *Hamiltonella*, whereas the uninfected line had an intermediate level of susceptibility (Fig. 2c; GLMM: *χ*^2^_3_ = 11.4, *p* = 0.009). In single infections, all *Fukatsuia* isolates reduced the susceptibility to *P. neoaphidis*, but there was significant variation between the isolates in the extent of this (Fig. 2d; GLMM: *χ*^2^_4_ = 82.79, *p* < 0.001). Isolate *F*_5_ provided the greatest protection when compared to the uninfected line, with isolates *F*_*1*_ and *F*_*3*_ also significantly protective in posthoc tests (Fig. 2d). None of the *Hamiltonella* isolates reduced susceptibility to *P. neoaphidis*, with some isolates actually increasing it and some variation between isolates (GLMM: *χ*^2^_4_ = 10.12, *p* = 0.04).

In co-infections, isolate of both species again affected the susceptibility to *P. neoaphidis*; however, there were again greater differences between isolates of *Fukatsuia* (*Hamiltonella*: GLMM: *χ*^2^_4_ = 34.54, *p* < 0.001; *Fukatsuia*: GLMM: *χ*^2^_4_ = 210.95, *p* < 0.001). There were also significant interactions between isolates of *Hamiltonella* and *Fukatsuia* (GLMM: *χ*^2^_16_ = 92.35, *p* < 0.001). This is most clearly illustrated by the *F*_1_ and *F*_2_ isolates (Fig. 2d): For *F*_2_, the presence of any of the *Hamiltonella* isolates did not have an effect on the aphid’s susceptibility to *P. neoaphidis*. In contrast, the identity of the *Hamiltonella* strain significantly affected *F*_1_’s ability to protect the aphid; in the presence of *H*_4,_ *F*_1_ provided a similar level of protection as in single infections, whereas in a co-infection with *H*_1_ it did no longer protect.

### Fecundity on a “specialist” and a “generalist” host plant

**Fig. 3:**
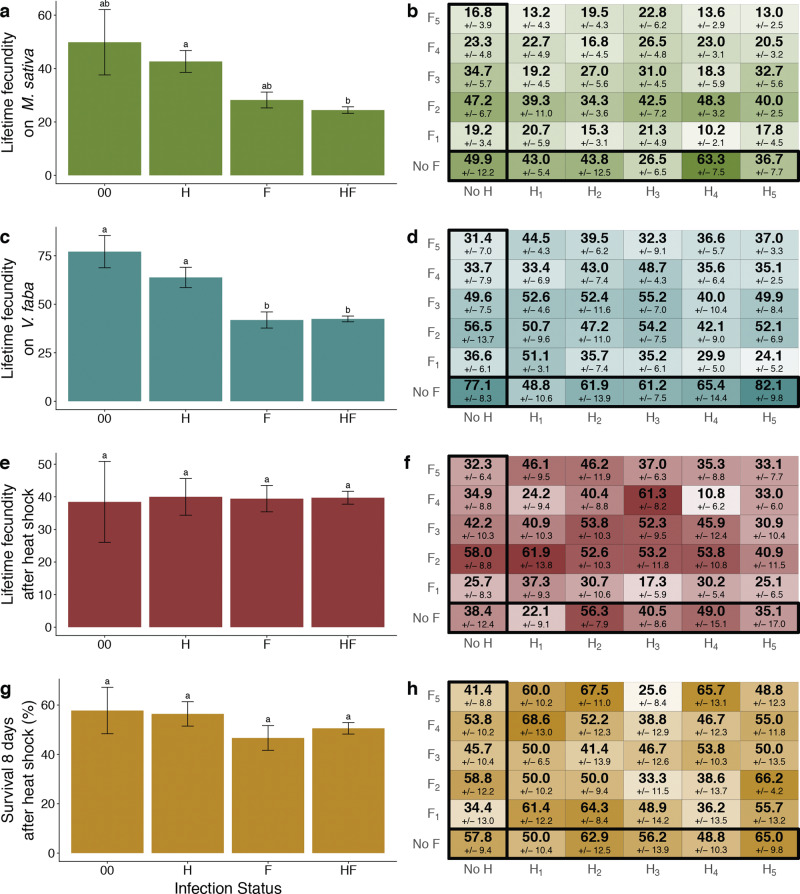
Fecundity measures and survival after heat shock, by infection status and by individual aphid line (± standard error). **a-b** Lifetime fecundity on original host plant *Medicago sativa*, *N* = 6 for all lines; (**c-d)** lifetime fecundity on laboratory host plant *Vicia faba*; (**e-f**) fecundity after heat shock; and (**g-h**) survival after heat shock. Seven to eleven replicates (mean 8.3) were conducted for each aphid line and heat treatment. 00 = uninfected aphid line; H = *Hamiltonella defensa*; F = *Fukatsuia symbiotica*; HF = co-infection. For tables of individual lines, shade of colour is relative to value in cell (darker = more fecund, or higher survival rate), note that while there is substantial variation between lines, for some lines there are also relatively large standard errors.

Similar patterns of fecundity were seen for both host plants, the ‘specialised’ host *M. sativa* and the ‘generalist’ host *V. faba*, although in general fecundity was higher on the latter (Fig. 3a and c; Pearson’s product-moment correlation: *t* = 5.59, d.f. = 34, *p* < 0.001, *r* = 0.69). Uninfected aphids and aphids with single infections of *Hamiltonella* on average had the most offspring on *V. faba*, with a similar trend on *M. sativa*. Those aphids co-infected with both species of symbiont or infected with only *Fukatsuia* produced the least offspring (Fig. 3a; *M. sativa* GLMM: *χ*^2^_3_ = 11.93, *p* = 0.008; Fig. 3c; *V. faba* LMM: *χ*^2^_3_ = 26.62, *p* < 0.001). The single isolates of both species varied in their effects on fecundity on *M. sativa* with greater variation among the *Fukatsuia* than among the *Hamiltonella* isolates (Fig. 3b; LMM: *Hamiltonella*: *χ*^2^_4_ = 10.57, *p* = 0.03; *Fukatsuia*: LMM: *χ*^2^_4_ = 20.67, *p* < 0.001). In contrast, there was no significant variation between isolates in single infections of either species on *V. faba* (Fig. 3d; LMM: *Hamiltonella*: *χ*^2^_4_ = 4.74, *p* = 0.32; *Fukatsuia*: *χ*^2^_4_ = 5.37, *p* = 0.25).

On both host plants, when aphids were co-infected with both *Hamiltonella* and *Fukatsuia*, the isolate of the former had no significant impact on fecundity (*V. faba*: LMM: *χ*^2^_4_ = 6.75, *p* = 0.15; *M. sativa*: LMM: *χ*^2^_4_ = 3.78, *p* = 0.44), but the isolate of the latter explained much of the variation (*V. faba*: LMM: *χ*^2^_4_ = 22.81, *p* < 0.001; *M. sativa*: LMM: *χ*^2^_4_ = 68.41, *p* < 0.001). Those *Fukatsuia* isolates that tended to reduce fecundity when infecting singly, also caused a reduction when in co-infections. The interaction of the isolates of the two species did not significantly impact on fecundity (*V. faba*: LMM: *χ*^2^_16_ = 10.18, *p* = 0.86; *M. sativa*: LMM: *χ*^2^_16_ = 15.44, *p* = 0.49).

### The effect of heat shock

On average, 51% of aphids survived heat shock (Fig. 3g), compared to controls where survival averaged 86% across different infections. Lifetime fecundity averaged 46.3 offspring in the control treatments (Fig. 3c – *V. faba* data) and 39.7 offspring in the heat treatments (Fig. 3e), showing an interaction of heat treatment and infection status (LMM: χ^2^_3_ = 17.63, p < 0.001). Both uninfected and *Hamiltonella* infected lines dropped markedly in reproductive output after being heat shocked, whereas lines infected or co-infected with *Fukatsuia* showed almost no change (Fig S2).

### Heat shock survival and recovery

Infection status did not significantly affect survival eight days after being heat shocked (Fig. 3g; GLMM; *χ*^2^_3_ = 2.46, *p* = 0.48) or lifetime fecundity after being heat shocked (Fig. 3e; LMM: *χ*^2^_3_ = 0.02, *p* = 0.99). Across the single infections, neither the *Hamiltonella* isolate nor the *Fukatsuia* isolate caused a significant difference in fecundity after heat shock (Fig. 3f; *Hamiltonella*: LMM: *χ*^2^_4_ = 4.06, *p* = 0.40; *Fukatsuia*: LMM: *χ*^2^_4_ = 7.53, *p* = 0.11), nor did any differ from the uninfected line. Similarly, survival eight days after heat shock was not affected by the individual *Hamiltonella* isolate (Fig. 3h; GLMM: *χ*^2^_4_ = 2.97, *p* = 0.56) or the *Fukatsuia* isolate (Fig. 3h; GLMM: *χ*^2^_4_ = 6.00, *p* = 0.20).

In co-infections, the two species of symbiont affected survival and fecundity in contrasting ways. The isolate of *Hamiltonella* caused significant variation in survival (LMM: *χ*^2^_4_ = 21.64, *p* < 0.001) but not subsequent reproduction (LMM: *χ*^2^_4_ = 4.61, *p* = 0.33), whereas the isolate of *Fukatsuia* did not affect survival (LMM: *χ*^2^_4_ = 0.69, *p* = 0.95) but was important for the survivors’ reproduction (LMM: *χ*^2^_4_ = 17.17, *p* = 0.002). The interaction between isolates did not impact on either the survival of co-infected aphids (LMM: *χ*^2^_16_ = 18.80, *p* = 0.28), or on their subsequent fecundity (LMM: *χ*^2^_16_ = 17.22, *p* = 0.37).

## Discussion

To our knowledge, this is the first study to demonstrate genotype-by-genotype (G×G) interactions between species of endosymbionts harboured by insects and the first to quantify the relative importance of G×G interactions relative to species interactions. G×G interactions are likely to alter the evolutionary dynamics of host populations as selection acts within the microbiome as well as on the host phenotype, which is determined by the genotypes of host and both microbes. At the species level, aphid lines co-infected with any isolate of both *Hamiltonella* and *Fukatsuia* symbionts showed an overall average usually reflecting that of *Fukatsuia* in single infections, apart from assays testing susceptibility to parasitoids, where *Hamiltonella* was the dominant species. Therefore, if we ignore isolate-level variation, co-infected lines usually benefit from higher protection against parasitoids and pathogens but suffer from lower fecundity as *Hamiltonella* is unable to rescue the large fecundity costs of harbouring *Fukatsuia*. However, when viewing the 36 aphid lines individually, the overall patterns are more variable as different isolates of the same symbiont species differ in the phenotype they confer. In each of the ecological challenges we posed aphids, and significantly so against pathogens and parasitoids, the phenotype displayed by the host was dependent on the specific combination of infecting symbiont isolates, not just the presence of both symbiont species. Despite the prevalence of co-infections in pea aphid populations worldwide [26, 38, 57], it is rarely beneficial for a host to harbour more than one facultative symbiont in any one ecological scenario (Text S8).

One caveat in our dataset is the presence of *Spiroplasma* in all lines harbouring isolate *F*_5_. We present the results of the full dataset as the removal of these lines from our analyses did not qualitatively impact the results (Text S7; Fig. S3). *Spiroplasma* can confer fungal protection to some genotypes of *A. pisum* [50], but can also increase susceptibility in single infections when compared to co-infections with *Fukatsuia* [46] and our analyses suggest that it has no effect here (Text S6). Importantly, the relative roles of species, isolates, and their interactions remain very similar in the full and reduced datasets (Fig. 1 vs Fig. S3).

To our surprise, the fact that two species co-existed did not predict a phenotype for any of the traits we measured, whereas the genetic identity in these interactions did, especially when considering interactions with natural enemies. If this is a general pattern in biological systems then species interactions in ecological communities ought to be investigated at the genotypic level instead of the species level, as is usual practice. Here, genetic interactions explained a considerable 17–52% of the phenotypic variation, and 47 to almost 100% when isolate identity overall was included (Fig. 1). The recent meta-analysis by Des Roches et al. [21] found that ecological processes are often just as or more strongly affected by variation within a species than by the presence of a particular species. Our data suggest that this balance can also be dramatically tipped in favour of G×G interactions relative to species interactions. We thus emphasise the need to quantify the contribution of G×G interactions as loss of genetic diversity may have wider ecological implications than is currently assumed. Here, the relative importance of G×G interactions was greater for resistance to natural enemies than for heat tolerance and fecundity, a core life history trait. Des Roches et al. [21] reported that genetic identity affected indirect ecological effects more strongly than direct effects; in particular community composition was often more strongly affected by particular genotypes than by the removal or replacement of a species. This indicates that traits that are involved in specific species interactions more frequently show greater genetic diversity and specificity in interactions. This might be the result of coevolutionary dynamics, where genetic specificity between host-associated defensive symbionts and the host’s natural enemies leads to frequency-dependent selection [58, 59].

More specifically, in host-microbe interactions the importance of genetic identity has previously been recognised, for example in the vertebrate gut [60, 61] and the pea aphid [51, 62]. Yet, interspecific microbe-microbe G×G interactions within hosts have not been considered at the scale investigated in our study. Within just one bacterial species, isolate × isolate interactions have been shown for a few model organisms, such as competing strains of *Vibrio fischeri* that colonise light organ crypts of the squid *Euprymna scolopes* [15] and *Hamiltonella* isolates co-infecting the pea aphid [17]. Most studies investigating interspecific interactions between symbionts infer patterns based on results from just one genotype of each infecting partner on one host genotype [63–65]. There is now growing evidence demonstrating versatility within a symbiont species’ protective repertoire [42, 46, 66, 67]. This versatility may be a result of genetic variation across strains, some of which is due to a variety of mobile genetic elements [45, 57, 68], for example variation in resistance to the parasitoid *A. ervi* is due to different variants of the phage APSE in the *Hamiltonella* genome [47, 48]. There is also evidence of significant G×G interactions between host and symbionts [14], or parasitoid or pathogen genotype [51, 69].

One important question is how co-infections are maintained within a host lineage and how stable they are. While our data cannot answer this directly, the analysis of how a co-infected aphid fares compared to the two singly infected counterparts allows some inferences. On average, co-infections were less beneficial to the host than the ‘best’ single counterpart for fecundity and equally beneficial for all other traits (Text S8; Fig. S4). Co-infected hosts were generally more resistant to natural enemies than the “worst” counterpart and no worse off for other traits. This indicates that in a stable environment there is unlikely selection for co-infections but that there are often scenarios that allow the worse partner to hitchhike with the better partner as there are no additional costs. However, the temporal variability of selective pressures in the field suggests that the help of different symbionts at different times may maintain co-infections, since across traits it varies which partner is best (illustrated in Fig. S5). Such selection could lead to fluctuating frequencies of aphid-symbiont communities, or to selection within the host where one symbiont might be eliminated through competitive interactions, regulation by the host or reduced vertical transmission. This may go some way to explain the variable results observed in other studies on co-infections [14, 17]. It is also important to note that only one of the aphid-symbiont combinations in this study is natural and the specific combinations employed here might not be maintained in a natural population.

Mechanistically, the diversity of phenotypes resulting from co-infections between *Fukatsuia* and *Hamiltonella* is difficult to explain. There are clear limiting factors to co-infections such as increased resource use, but also potential avenues for co-existence and complementarity. In some cases, the density of a symbiont is affected by the presence of another [16, 18] and this in turn might affect its phenotypic effects. We cannot rule this out, but it would not explain some aspects of our data. For instance, line *H*_4_*F*_1_ is more protective than both its single counterparts, indicating some protective complementarity between the two symbionts, although note that Patel et al. [68] suggest that *metabolic* complementarity is unlikely to be the basis of *Fukatsuia* frequently co-infecting aphids alongside *Hamiltonella*. It is also possible that isolates rapidly respond to their specific environmental scenario by altering their relative densities.

Community context is paramount to make predictions about the outcomes of microbial interactions [70]. When extrapolating function there needs to be more consideration of the specific community at both the species and the genotype level. We illustrate that even within a single host genotype the effects of harbouring multiple bacterial symbionts can be highly specific. As the symbionts are typically co-inherited along with host genes, selection on both the host phenotype and symbiont interactions within the host will affect the evolutionary trajectory of the community. Similar patterns are likely in other communities where co-inheritance of closely associated species is common [25] but the eco-evolutionary consequences of G×G interactions in these systems are poorly understood.

## Supplementary information

Supplemental Material

README

Data_FungalChallenge

Data_HeatshockSurvivalXFecundity

Data_M.sativaFecundity

Data_ParasitoidChallenge

## Data Availability

All data generated or analysed during this study are included in the supplementary information files of this article.
