# Supplementary material for: Genetic identity and genotype × genotype interactions between symbionts outweigh species level effects in an insect microbiome
Source: ISME J. 2021 Mar 12;15(9):2537–46. doi: 10.1038/s41396-021-00943-9 (PMC8397793; doi:10.1038/s41396-021-00943-9)
Supplement: Supplementary file 1 — Supplemental Material [file 41396_2021_943_MOESM1_ESM.pdf]

Supplementary material for:

Genetic identity and genotype × genotype interactions between symbionts outweigh species level effects in an insect microbiome.

Smee, M.R., Raines, S.A. and Ferrari, J.

Table of Contents

Materials and methods ***2***

S1 - Creation and maintenance of aphid lines **2**

Table S1 – The 36 manipulated aphid lines used in this study.. 2

Table S2 – Natural aphid lines used to create the 36 manipulated lines used in the current study. 2

S2 - Details of DNA extraction, primers and PCR protocols **3**

Table S3 - Primer sequences for PCR reactions. 4

S3 - Susceptibility to the parasitoid *Aphidius ervi* **4**

S4 - Susceptibility to the fungal pathogen *Pandora neoaphidis* **5**

S5 - Data analysis **5**

Table S4 - Overview of the statistical tests used for each ecological scenario 5

S6 - Loss and re-injection of *F. symbiotica* from lines H_2F_4 and H_2F_5 .... **8**

Figure S1 – The susceptibility of co-infected aphid lines H_2F_4 and H_2F_5 to the fungal pathogen *P. neoaphidis* before, during and after the loss of *F. symbiotica*. 8

Figure S2 – Comparison of fecundity after heat shock and general fecundity **9**

S7 - Summary of effects of *Spiroplasma* infection in lines with the *Fukatsuia* isolate F_5 .... **10**

Table S5 – Overview of statistical tests used on data not including the six lines also harbouring *Spiroplasma*. 10

Figure S3 – The proportion of the deviance explained in generalised linear models (GLM). As in Figure 1, but without *Spiroplasma* lines included 12

S8 - How frequent are synergies or antagonisms in co-infections? **13**

Figure S4 - Performance of co-infections when compared to their 'best' or 'worst' single counterpart, across all ecological scenarios........ 14

Figure S5 – Line by line expansion of Figure S4. .... **16**

References **17**

Materials and methods

S1 - Creation and maintenance of aphid lines

The curing process involved feeding aphids for three days on single leaf pairs with their petioles placed in an antibiotic cocktail of 1% Ampicillin, 0.5% Gentamicin and 0.5% Cefotaxime [1]. Any survivors were kept, allowed to reproduce, and their first 10 offspring discarded. The next few offspring were kept individually until they reproduced, at which point adults were tested for the absence of symbionts using methods described in Text S2, and cured lines were kept.

Table S1 – The 36 manipulated aphid lines used in this study. The uninfected line is shaded in dark grey, and singly infected lines are shaded in light grey. All lines were created by microinjection from the donor aphid (see Table S2 for details of natural lines). All donor lines were collected from *Medicago sativa*. Lines carrying strain F5 also carried *Spiroplasma*.

217	**F_5**	**H_1F_5**	**H_2F_5**	**H_3F_5**	**H_4F_5**	**H_5F_5**
211	**F_4**	**H_1F_4**	**H_2F_4**	**H_3F_4**	**H_4F_4**	**H_5F_4**
236	**F_3**	**H_1F_3**	**H_2F_3**	**H_3F_3**	**H_4F_3**	**H_5F_3**
238	**F_2**	**H_1F_2**	**H_2F_2**	**H_3F_2**	**H_4F_2**	**H_5F_2**
218	**F_1**	**H_1F_1**	**H_2F_1**	**H_3F_1**	**H_4F_1**	**H_5F_1**
	Uninfected	**H_1**	**H_2**	**H_3**	**H_4**	**H_5**
Donor lines		218	238	236	207	216

Table S2 – Natural aphid lines used to create the 36 manipulated lines used in the current study. All lines were collected from *Medicago sativa*.

In Current Study	Aphid Clone	Symbiont(s)	Site Collected	Date Collected
H_1 and F_1	218	*Hamiltonella* & *Fukatsuia*	Eling	28th May 2010
H_2 and F_2	238	*Hamiltonella* & *Fukatsuia*	Beaconsfield	25th May 2010
H_3 and F_3	236	*Hamiltonella* & *Fukatsuia*	Beaconsfield	25th May 2010
H_4	207	*Hamiltonella*	Lincoln	31st August 2012
H_5	216	*Hamiltonella*	Lincoln	31st August 2012
F_4	211	*Fukatsuia*	Eling	28th May 2010
F_5	217	*Fukatsuia* & *Spiroplasma*	Eling	28th May 2010

Five aphid lines infected with *Hamiltonella*, and five infected with *Fukatsuia* were used as donors (Table S1). To create experimental lines H_1 and F_1 (Table S1), the original aphid line 218 was cured from one symbiont only. To obtain all other experimental lines, microinjection of haemolymph was used to transfer the donor's symbiont into the cured background of line 218. All lines were established within six months of each other, and experiments only started eight generations after the final line was created. To ensure no contamination with other aphid lines, the aphid genotype and symbiont status were regularly confirmed using microsatellite markers and diagnostic PCR checks [2].

We obtained genome sequences from all ten isolates of symbionts, which confirmed that all five isolates of each species cluster with other known strains of that species based on 550 single copy orthologous genes (SICOs). The *Hamiltonella* isolates used here fall into two clusters, with H_1, H_2 and H_3 clustering together, and H_4 and H_5 forming a second clade. Within *Fukatsuia* and within the two *Hamiltonella* clades, there was little variation in the SICOs, but substantial variation in mobile genetic elements including in insertion sequences, plasmids and phage islands. Notably, H_1-H_3 contained the APSE variant APSE-1, and H_4 and H_5 contained APSE-2.

S2 - Details of DNA extraction, primers and PCR protocols

Surviving aphids were kept for a minimum of eight generations and tested frequently using diagnostic PCR. Other known symbionts of the pea aphid were also tested for but not found: *Regiella insecticola*, *S. symbiotica*, *Spiroplasma* sp., *Rickettsia* sp. and *Rickettsiella viridis*.

Aphids were homogenised in a 200 µl 5% Chelex solution made in distilled water. 10 µl of proteinase K (Promega, 10 mg/ml) was added per sample, and samples were incubated overnight at 56°C to facilitate digestion. They were then 'boiled' at 100°C for ten minutes before being centrifuged at 13,000 rpm for 3 minutes and the supernatant containing the DNA pipetted into a clean 1.5 ml Eppendorf tube which was stored at -20°C until use, or used immediately.

The PCR mix comprised 6.25 µl BioMix (Bioline), 0.1 µl (20 µM) of forward and 0.1 µl (20 µM) reverse primer (Table S3), 5.55 µl distilled water and 1.0 µl sample DNA. The PCR reaction for all symbionts except *Rickettsia* was performed at 94°C for 2 minutes, followed by 35 cycles of: 94°C for 30 seconds, 55°C for 30 seconds and 72°C for 1 minute. It concluded with 6 minutes at 72°C and then cooled the sample to 4°C indefinitely. The PCR reaction for *Rickettsia* also started with 2 minutes at 94°C, followed

by 10 cycles of: 94°C for 1 min, 65 - 55°C for 1 min in 1°C steps per cycle, and 72°C for 2 minutes, and then 25 cycles of: 94°C for 1 minute, 55°C for 1 minute and 72°C for 2 minutes. A final step of 6 minutes at 72°C and then the samples were cooled to 4°C indefinitely. PCR products were run on a 1% agarose gel and the presence of a band confirmed the presence of the symbiont.

Table S3 - Primer sequences for PCR reactions.

Symbiont	Forward	Sequence	Reverse	Sequence	Reference
Hamiltonella	10F	5'-AGTTTGATCATGGCTCAGATTG-3'	T419R	5'-AAATGGTATTSGCATTTATCG-3'	Ferrari *et al*, 2012
Fukatsuia	10F	5'-AGTTTGATCATGGCTCAGATTG-3'	X420R	5'-GCAACACTCTTTGCATTGCT-3'	Ferrari *et al*, 2012
Regiella	10F	5'-AGTTTGATCATGGCTCAGATTG-3'	U433R	5'-GGTAACGTCAATCGATAAGCA-3'	Ferrari *et al*, 2012
Serratia	10F	5'-AGTTTGATCATGGCTCAGATTG-3'	R443R	5'-CTTCTGCGAGTAACGTCAATG-3'	Ferrari *et al*, 2012
Spiroplasma	10F	5'-AGTTTGATCATGGCTCAGATTG-3'	TKSSsp	5'-TAGCCGTGGCTTTCTGGTAA-3'	Fukatsu & Nikoh, 2000
Rickettsia sp.	16SA1	5'-AGAGTTTGATCMTGGCTCAG-3'	Rick16SR	5'-CATCCATCAGCGATAAATCTTTC-3'	Fukatsu *et al*, 2001
Rickettsiella viridis	RCL_16S -211F	5'-GGGCCTTGCGCTCTAGGT-3'	RCL_16S -470R	5'-TGGGTACCGTCACAGTAATCGA-3'	Tsuchida *et al*, 2010

S3 - Susceptibility to the parasitoid Aphidius ervi

To determine how different combinations of symbiont isolates might affect this protection, groups of 30 three- to four-day-old aphids were exposed to individual *A. ervi* females as detailed in Heyworth and Ferrari [3]. Aphids were placed on *V. faba* plants in a single pot, enclosed by a vented, clear plastic cage. A single female wasp that had emerged up to 24 hours earlier was introduced and allowed to forage for aphids for nine hours. Ten days after exposure the number of 'mummies' formed by parasitoid larvae developing inside any successfully parasitised aphids were counted, as well as the number of surviving non-parasitised aphids. Parasitoid wasps have been shown to elicit different behaviours when ovipositing in aphids harbouring different symbionts, but usually this is when presented with a choice, rather than the no-choice assay employed here, so we interpret that any differences found are due to physiological resistance [4–6].

S4 - Susceptibility to the fungal pathogen Pandora neoaphidis

As detailed in Heyworth and Ferrari [3], groups of 20 ten-day-old apterous aphids were exposed to sporulating cadavers. Each group of 20 aphids was placed in a small plastic tube with an opening one end (height 50 mm, diameter 15 mm). Pairs of cadavers that had been generated from a symbiont-free aphid line and kept in the fridge at 4°C for no more than a month were placed on small pieces of damp filter paper. These were then suspended upside down from the lids of Petri dishes and left overnight at 20°C and high humidity to start the fungus sporulating. At the start of the 90-minute experiment, these damp filter papers with sporulating cadavers were suspended over the tubes of aphids to create a spore shower. Cadavers were rotated among replicates throughout the experiment to account for differing sporulating efficiencies. At the end of the 90 minutes, each group of 20 aphids was placed on a fresh two-week-old *V. faba* plant. After ten days plants were checked regularly for infected and sporulating aphid cadavers, and after a total of two weeks the remaining aphids left alive were counted. Note that during the course of the study diagnostic PCRs showed that *Fukatsuia* had been lost from lines H_2F_4 and H_2F_5. These lines were re-created by microinjection and tested again for susceptibility to *P. neoaphidis.* In our analysis we use the data from experimental blocks before *Fukatsuia* was lost, and after the lines were re-created as they showed a consistent level of susceptibility (Figure S1 and Text S6).

S5 - Data analysis

Table S4 - Overview of the statistical tests used for each ecological scenario. Model abbreviations are: GLMM: Generalised linear mixed model; LMM: Linear mixed model; GLM: Generalised linear model. Parasitoids: Susceptibility to *Aphidius ervi*, Fungus: Susceptibility to *Pandora neoaphidis*, *Medicago*: Fecundity on *Medicago sativa*, *Vicia*: Fecundity on *Vicia faba*, Heat shock Fecundity: Lifetime fecundity after heat shock, Heat shock Survival: seven-day survival after heat shock.

Experiment	Model	Data used	Statistics	Notes
**Parasitoids**	Infection	All data, at level of species.	GLMM (binomial)	+1 to response variable
	H strains	Five single H lines only	GLMM (binomial)	+1 to response variable
	F strains	Five single F lines only	GLMM (binomial)	+1 to response variable
	Doubles only	25 co-infected lines only	GLMM (binomial)	+1 to response variable and BOBYQA optimiser
	Variance model	All data	GLM (quasi-binomial)	+1 to response variable

	Co-infection vs. 'best' or 'worst' single	Difference between co-infection and mean single counterpart.	LMM	
**Fungus**	Infection	All data, at level of species.	GLMM (binomial)	+1 to response variable
	H strains	Five single H lines only	GLMM (binomial)	+1 to response variable
	F strains	Five single F lines only	GLMM (binomial)	+1 to response variable
	Doubles only	25 co-infected lines only	GLMM (binomial)	+1 to response variable and BOBYQA optimiser
	Variance model	All data	GLM (quasi-binomial)	+1 to response variable
	Co-infection vs. 'best' or 'worst' single	Difference between co-infection and mean single counterpart.	LMM	
Medicago	Infection	All data, at level of species.	GLMM (Poisson)	
	H strains	Five single H lines only	LMM	
	F strains	Five single F lines only	LMM	
	Doubles only	25 co-infected lines only	LMM	
	Variance model	All data	GLM (quasi-Poisson)	
	Co-infection vs. 'best' or 'worst' single	Difference between co-infection and mean single counterpart.	LMM	
Vicia	Infection	All data, at level of species.	LMM	
	H strains	Five single H lines only	LMM	
	F strains	Five single F lines only	LMM	
	Doubles only	25 co-infected lines only	LMM	
	Variance model	All data	GLM	
	Co-infection vs. 'best' or 'worst' single	Difference between co-infection and mean single counterpart.	LMM	
**Heat shock Fecundity**	Infection	All data, at level of species.	LMM	
	H strains	Five single H lines only	LMM	
	F strains	Five single F lines only	LMM	
	Doubles only	25 co-infected lines only	LMM	
	Variance model	All data	GLM	
	Co-infection vs. 'best' or 'worst' single	Difference between co-infection and mean single counterpart.	LMM	

**Heat shock Survival**	Infection	All data, at level of species.	GLMM (binomial)	
	H strains	Five single H lines only	GLMM (binomial)	Observation-level random effect used to counter overdispersion
	F strains	Five single F lines only	GLMM (binomial)	Observation-level random effect used to counter overdispersion
	Doubles only	25 co-infected lines only	LMM	Proportion as response
	Variance model	All data	GLM (quasi-binomial)	
	Co-infection vs. 'best' or 'worst' single	Difference between co-infection and mean single counterpart.	LMM	
**Heat shock vs. Controls**	Infection-level comparison	All data, at level of species.	LMM	

S5.1

Posthoc tests were carried out using Tukey's HSD test for multiple comparisons of means, with package 'multcomp' [7] at a significance level of $p < 0.05$. For two of the fecundity datasets (broad bean and heat shock), visual model checks confirmed that the residuals were normally distributed when using the raw data, so only linear mixed models were used. Due to an excess of zeros in the wasp and fungus datasets, we used the response variable cbind(successes + 1, failures + 1) (see Table S4).

S5.2

When analysing the set of 25 co-infected lines only, for the wasp and fungus datasets, it was necessary to increase the maximum number of function evaluations run by the model optimiser BOBYQA to enable the binomial GLMM models to converge. We also employed an observational-level random effect where necessary, to cope with over-dispersion in the data found by using the package 'blmeco' [8] (see Table S4).

S6 - Loss and re-injection of F. symbiotica from lines H_2F_4 and H_2F_5

During the course of the study diagnostic PCRs showed that *F. symbiotica* had been lost from lines H_2F_4 and H_2F_5. These lines were re-created by microinjection and tested again for susceptibility to *P. neoaphidis* as this was the only experiment affected. Interestingly, and despite the presence of *Spiroplasma* in line H_2F_5, susceptibility increased to the level of the single *H. defensa* infection during the period when *F. symbiotica* was lost, and once re-established susceptibility decreased again (Fig. S1).

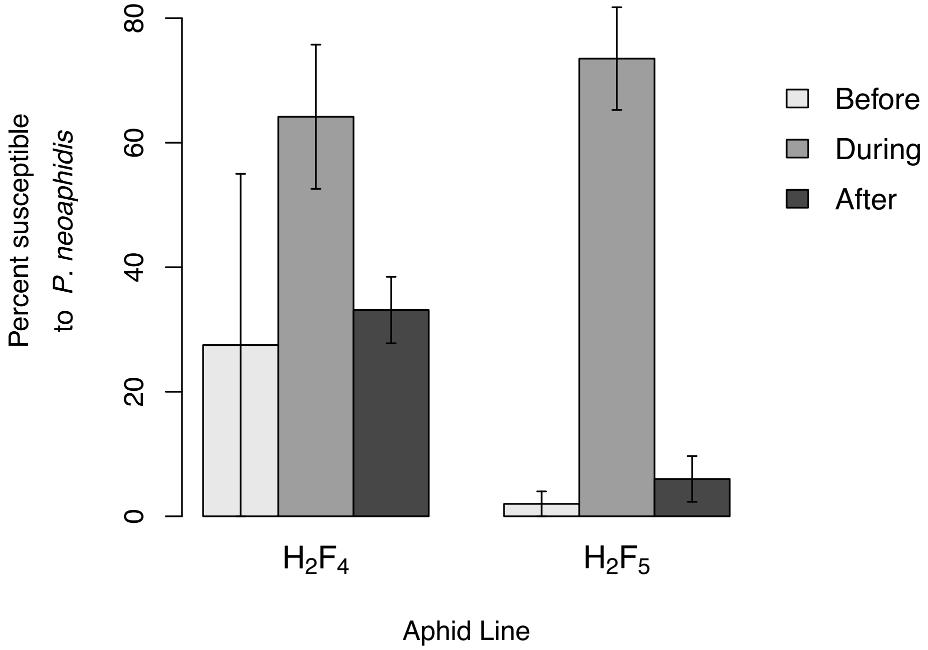

Figure S1 – The susceptibility of co-infected aphid lines H_2F_4 and H_2F_5 to the fungal pathogen *P. neoaphidis* before the loss of *F. symbiotica* (Before, light gray), during experiments whilst the symbiont was lost (During, mid-gray), and after re-establishment in the same aphid lines (After, dark gray). Means are shown, ± standard error.

Figure S2 – Comparison of fecundity after heat shock and general fecundity.

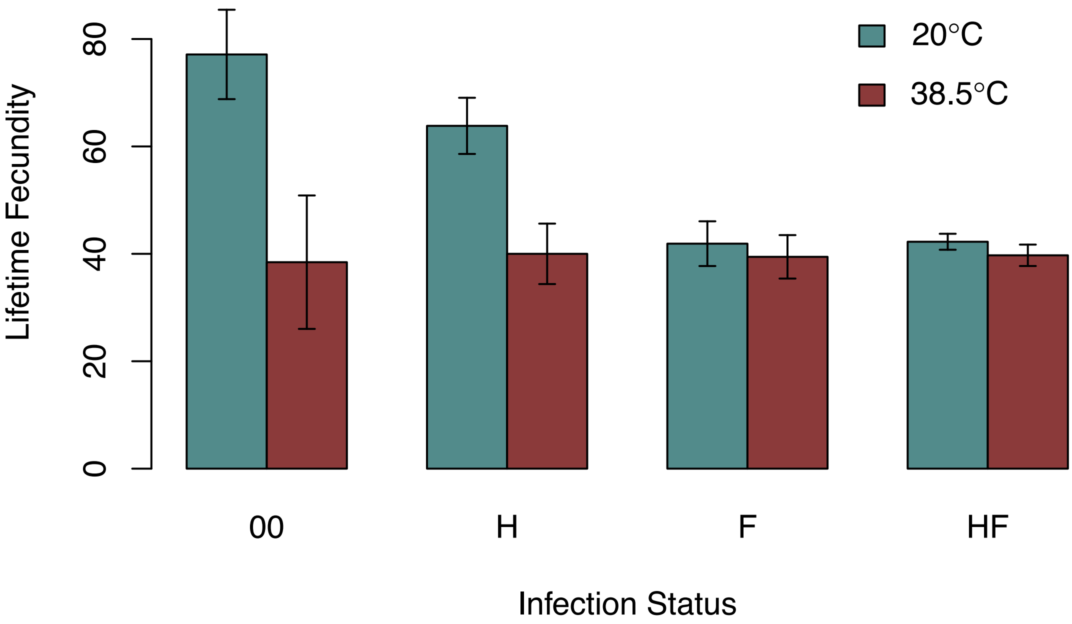

Figure S2 – Mean lifetime fecundity on laboratory host plant *Vicia faba* at 20ºC and after heat shock at 38.5ºC (± standard error). Data is as presented in Figure 3c and 3e in the main manuscript, but here plotted on the same axis to illustrate differences between the two conditions.

S7 - Summary of effects of Spiroplasma infection in lines with the Fukatsuia isolate F_5.

Table S5 – Overview of statistical tests used on data not including the six lines also harbouring *Spiroplasma* (isolate *F_5*). Model abbreviations are: GLMM: Generalised linear mixed model; LMM: Linear mixed model; GLM: Generalised linear model. Parasitoids: Susceptibility to *Aphidius ervi*, Fungus: Susceptibility to *Pandora neoaphidis*, *Medicago*: Fecundity on *Medicago sativa*, *Vicia*: Fecundity on *Vicia faba*, Heat shock (fecundity): Lifetime fecundity after heat shock, Heat shock (survival): seven-day survival after heat shock.

Experiment	Model	Data used	Statistics	Notes
**Parasitoids**	Infection	All data, at level of species.	GLMM (binomial)	+1 to response variable
	H strains	Five single H lines only	-	NA
	F strains	**Four** single F lines only	GLMM (binomial)	+1 to response variable
	Doubles only	**20** co-infected lines only	GLMM (binomial)	+1 to response variable and BOBYQA optimiser
	Variance model	All data	GLM (quasi-binomial)	+1 to response variable
**Fungus**	Infection	All data, at level of species.	GLMM (binomial)	+1 to response variable
	H strains	Five single H lines only	-	NA
	F strains	**Four** single F lines only	GLMM (binomial)	+1 to response variable
	Doubles only	**20** co-infected lines only	GLMM (binomial)	+1 to response variable
	Variance model	All data	GLM (quasi-binomial)	+1 to response variable
Medicago	Infection	All data, at level of species.	GLMM (Poisson)	
	H strains	Five single H lines only	-	NA
	F strains	**Four** single F lines only	LMM	
	Doubles only	**20** co-infected lines only	LMM	
	Variance model	All data	GLM (quasi-Poisson)	
Vicia	Infection	All data, at level of species.	LMM	
	H strains	Five single H lines only	-	NA
	F strains	**Four** single F lines only	LMM	
	Doubles only	**20** co-infected lines only	LMM	
	Variance model	All data	GLM	
**Heat shock Fecundity**	Infection	All data, at level of species.	LMM	
	H strains	Five single H lines only	-	NA

	F strains	**Four** single F lines only	LMM	
	Doubles only	**20** co-infected lines only	LMM	
	Variance model	All data	GLM	
**Heat shock Survival**	Infection	All data, at level of species.	GLMM (binomial)	
	H strains	Five single H lines only	-	NA
	F strains	**Four** single F lines only	GLMM (binomial)	Observation-level random effect used to counter overdispersion
	Doubles only	**20** co-infected lines only	LMM	Proportion as response
	Variance model	All data	GLM (quasi-binomial)	
**Heat shock vs. Controls**	Infection-level comparison	All data, at level of species.	LMM	

In general, the results of analyses not including the aphid lines also harbouring *Spiroplasma* (any line including F_5) gave the same or very similar results to the whole dataset. The main discrepancy is the loss of significance of *Fukatsuia* isolate in the fungal dataset, when F_5 lines are not included (Fig. 1 & S3). There is also a loss of significance in the parasitoid dataset for the original 'infection' model, from $p = 0.04$ to $p = 0.11$, possibly because the F_5 lines in general are more susceptible and therefore their removal leans the data further towards highly protective. Even so, there is still a significant interaction of symbiont isolates so the importance of individual isolates is not lost. Importantly, the relative roles of species, isolate and their interactions remain very similar in the full and reduced datasets.

In addition, the data presented in Figure S1 suggest that susceptibility to the fungal pathogen is a direct result of the presence of the infecting *Fukatsuia* strain, and that *Spiroplasma* plays no direct role in protecting these lines against fungal infection, despite previous studies showing that isolates of *Spiroplasma* may protect aphids from *P. neoaphidis* [9].

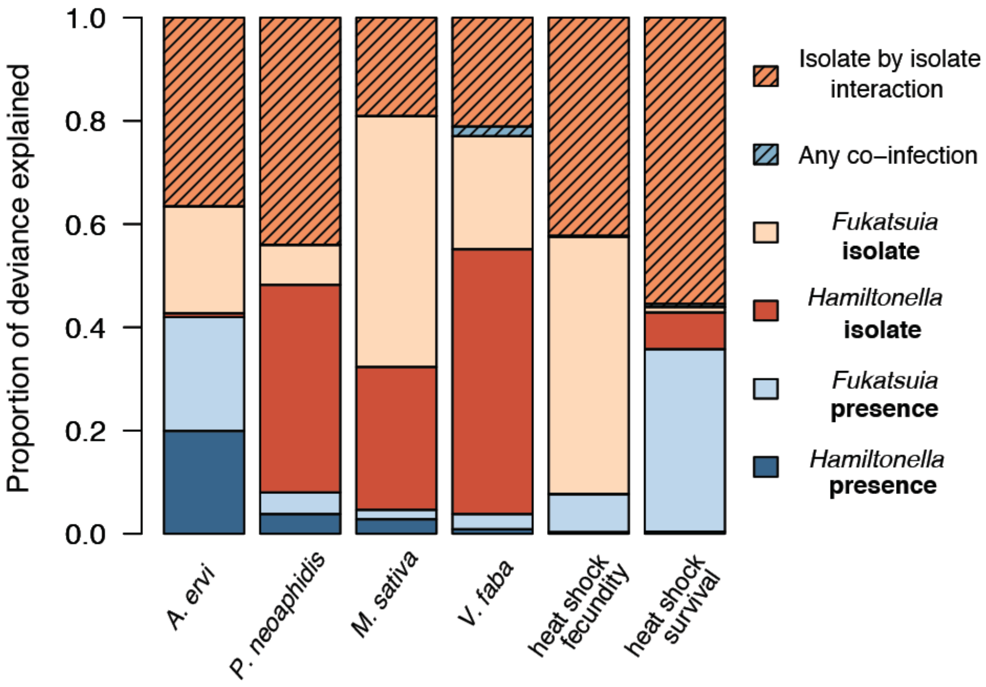

Figure S3 – The proportion of the deviance in generalised linear models (GLMs) for each experimental assay explained by just the presence of either of the two symbiont species, the individual isolates, the occurrence of any co-infection, and the co-infection between specific isolates. The analysis includes all aphid lines except those harbouring the F_5 isolate, and hence *Spiroplasma* too (see Figure 1 for the same analysis including all data).

S8 – How frequent are synergies or antagonisms in co-infections?

A key question is how interactions between specific symbionts within a host affect the host's phenotype, and whether there are usually synergies where the benefit to the host increases compared to the benefit provided by only the 'best' symbiont in a given combination. Alternatively, this benefit might be reduced by the presence of a partner or there might be no change. For a symbiont that provides no or less of a benefit compared to the 'best' symbiont in a given association – the 'worst' symbiont – there are also three possible outcomes: it could be rescued by the presence of the 'better' symbiont; there might be no change; or there might be antagonisms such as competition for resources, leading to an even less fit host phenotype.

Analysis

For all 25 co-infected lines and all traits, we tested how the host phenotype was altered by the presence of a 'better' or 'worse' co-infecting partner. For the analysis, we extracted the value of each replicate for each co-infected line for any given trait, and then subtracted the corresponding 'best' mean value of its single counterparts for that trait. We did the same for the 'worst' single counterpart. In all cases, a result of zero implies that the phenotype of the co-infected aphid is the same as the single counterpart of that infecting pair of isolates. For both parasitoids and fungal pathogens, a higher susceptibility is a bad outcome for the aphid, whereas for fecundity and survival measures a higher value is a good outcome. Therefore, to make the results easier to visualise, for protection against parasitoids and fungal pathogens we multiplied the resulting difference by -1. Consequently, for all traits measured, the larger the value the better the co-infection performed in comparison to the single counterpart it is compared to. We performed linear mixed models (LMMs) for all datasets, with the difference as calculated above as the response, against just the intercept of the model. The specific co-infection and the experimental block were included as random factors. If the intercept was significantly different from zero, this indicated a significant difference between the performance of co-infected lines versus their 'best' or 'worst' counterparts. When comparing to the 'best' counterpart, if co-infections perform better this would indicate synergistic effects of the two symbionts, whereas if the 'best' single infection outperforms the co-infection this would indicate a cost of the second symbiont to the host. Conversely, when comparing to the 'worst' counterpart, if the co-infection performs better it would suggest that the better symbiont is able to rescue the host. Whereas, if the co-infection performs the same as the 'worst' counterpart then that isolate essentially acts as a parasite in this context.

Results and Discussion

We found that, on average, co-infections did not result in synergistic effects and the host benefited more by harbouring only the 'best' performing partner of the two isolates, but only significantly so for fecundity on either host plant (Fig. S4; LMMs: parasitoids: $t_{14} = 1.69$, $p = 0.11$; fungal pathogen: $t_9 = 1.11$, $p = 0.30$; *M. sativa*: $t_{17} = 5.98$, $p < 0.001$; *V. faba*: $t_8 = 4.67$, $p = 0.002$; heat shock fecundity: $t_5 = 1.85$, $p = 0.12$; heat shock survival: $t_4 = 0.79$, $p = 0.47$). For fecundity, there was thus a cost to the aphid of harbouring a co-infection while for the other traits the benefit of the co-infection was equal to that of the 'best' single isolate. Yet, when confronted with the parasitoid or fungal pathogen, hosts benefited more on average from harbouring a co-infection than just the 'worst' single isolate (Fig. S4; LMMs: parasitoids: $t_{25} = 3.95$, $p < 0.001$; fungal pathogen: $t_9 = 4.69$, $p = 0.001$; *M. sativa*: $t_4 = 0.98$, $p = 0.38$; *V. faba*: $t_4 = 0.47$, $p = 0.66$; heat shock fecundity: $t_5 = 1.90$, $p = 0.12$; heat shock survival: $t_5 = 0.72$, $p = 0.51$). This suggests that, for these traits, the more protective isolate can rescue the host from the 'worst' isolate in a co-infection. For a line-by-line expansion of this analysis, see Fig. S5.

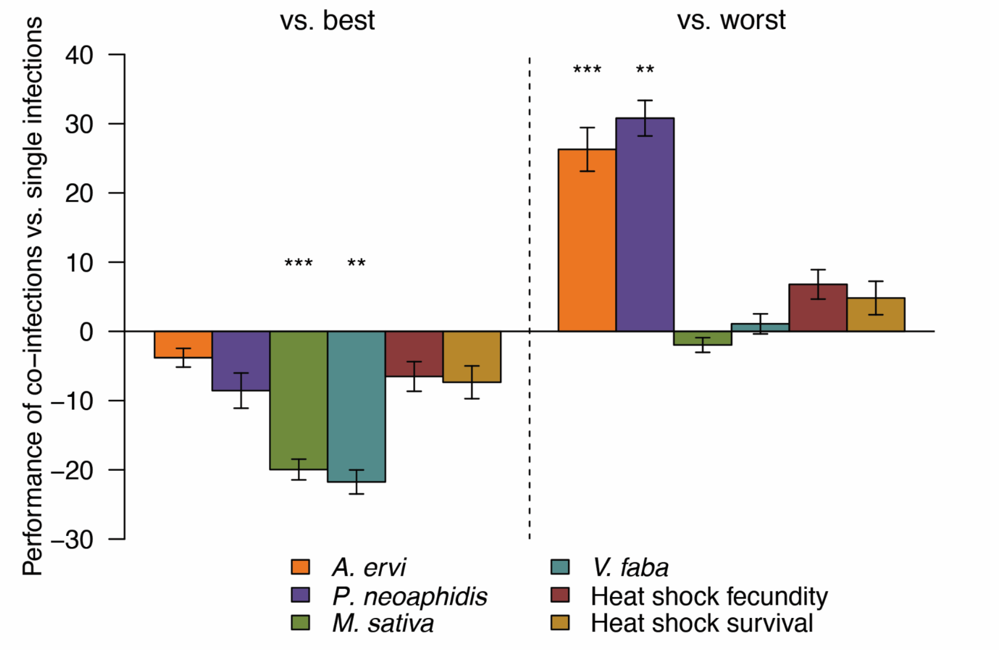

Figure S4– Performance of co-infections when compared to their 'best' or 'worst' single counterpart, across all ecological scenarios. Values are calculated for each of the 25 co-infected aphid lines by subtracting the mean value (from grids in Figures 2 and 3) of the 'best' or 'worst' infecting symbiont in a single infection from each replicate of the corresponding co-infection. For ease of viewing, values from parasitoid wasps and the fungal pathogen were multiplied by -1, so for all scenarios the larger the value the better the co-infection performed compared to the single infection. In all cases, a result of 0 implies that the co-infection performs the same as the single counterpart of interest from that infecting pair of isolates. Significance differences from zero are shown as: ** p < 0.01; *** p < 0.001. See Figure S5 for a line-by-line expansion of this analysis.

We have demonstrated that synergies between the co-infecting symbionts are rare in any given ecological scenario and as such, it is unlikely that there would be selection for co-infected aphids in a stable environment. In fact, co-infections are likely to be selected against in a benign environment as the fecundity of the co-infection is usually reduced compared to the 'best' corresponding single infection. However, when natural enemies are present, a co-infected aphid performs on average as well as the 'best' single partner, and there is thus the potential for the 'worse' partner hitchhiking along as has been suggested for *Fukatsuia* in North American pea aphid populations [10].

The relative performance of co-infected lines compared to their singly infected counterparts may go some way to explain the variable results observed in other studies on co-infections [11, 12]. The pattern observed here suggests that in a stable environment a singly infected aphid would outperform a co-infected one, but the temporal variability of selective pressures in the field suggests that the help of different symbionts at different times may maintain co-infections since across traits, it varies which partner is best (illustrated in Fig. S5).

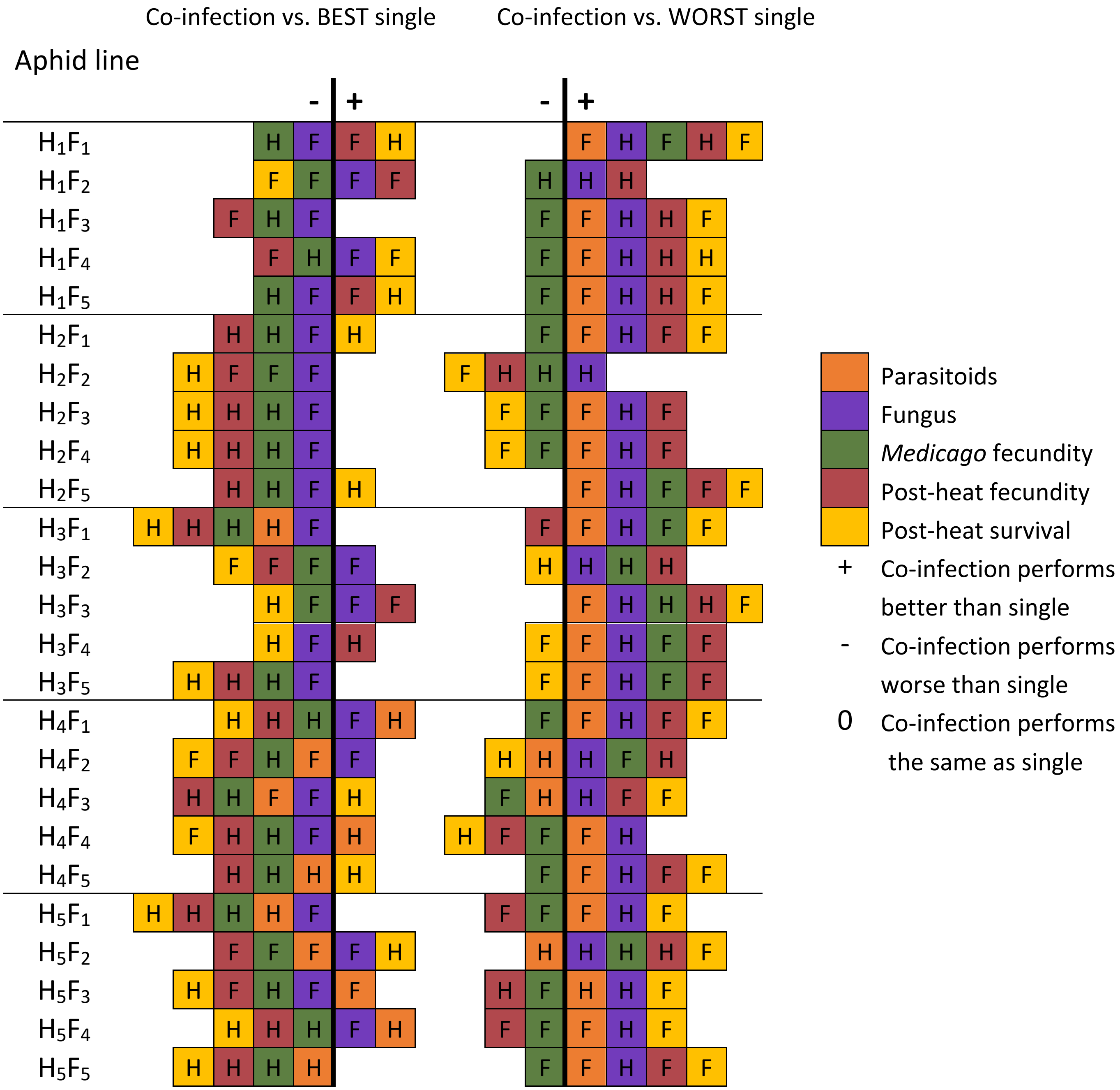

Figure S5 – Line by line account of how each co-infection performs compared to its 'best' and 'worst' single counterparts. An expansion of Figure S4. Values were calculated for each of the 25 co-infected aphid lines by subtracting the mean value (from grids in Figures 2 and 3) of the 'best' or 'worst' infecting symbiont in a single infection from the mean value of the corresponding co-infection. If the co-infection performs equal to the single infection, no colour block is added. If the co-infection performs better, a colour block is added on the '+' side, as indicated. Alternatively, if the co-infection performs worse, then a colour block is added on the '–' side. For each added block, the letter 'H' denotes that *Hamiltonella* was the 'best' or 'worst' in that instance, and the letter 'F' denotes that *Fukatsuia* was. For almost all lines in the comparison to the 'best' counterpart, there are more blocks on the '–' side, indicating the co-infections are rarely better for a host than the 'best' single counterpart. Conversely, co-infections are usually better than the 'worst' single infection.

References

1. McLean AHC, van Asch M, Ferrari J, Godfray HCJ. Effects of bacterial secondary symbionts on host plant use in pea aphids. *Proc R Soc B-Biol Sci* 2011; **278**: 760–766.
2. Ferrari J, Via S, Godfray HCJ. Population differentiation and genetic variation in performance on eight hosts in the pea aphid complex. *Evolution* 2008; **62**: 2508–2524.
3. Heyworth ER, Ferrari J. A facultative endosymbiont in aphids can provide diverse ecological benefits. *J Evol Biol* 2015; **28**: 1753–1760.
4. Henter HJ, Via S. The potential for coevolution in a host-parasitoid system. I. Genetic variation within an aphid population in susceptibility to a parasitic wasp. *Evolution* 1995; **49**: 427–438.
5. Łukasik P, Dawid MA, Ferrari J, Godfray HCJ. The diversity and fitness effects of infection with facultative endosymbionts in the grain aphid, Sitobion avenae. *Oecologia* 2013; **173**: 985–996.
6. Oliver KM, Noge K, Huang EM, Campos JM, Becerra JX, Hunter MS. Parasitic wasp responses to symbiont-based defense in aphids. *BMC Biol* 2012; **10**: 11.
7. Hothorn T, Bretz F, Westfall P. Simultaneous Inference in General Parametric Models. *Biom J* 2008; **50**: 346–363.
8. Korner-Nievergelt F, Roth T, Felten S von, Guélat J, Almasi B, Korner-Nievergelt P. Bayesian Data Analysis in Ecology Using Linear Models with R, BUGS, and Stan. 2015. Academic Press.
9. Łukasik P, van Asch M, Guo H, Ferrari J, Charles J. Godfray H. Unrelated facultative endosymbionts protect aphids against a fungal pathogen. *Ecol Lett* 2013; **16**: 214–218.
10. Doremus MR, Oliver KM. Aphid heritable symbiont exploits defensive mutualism. *Appl Environ Microbiol* 2017; **83**: e03276-16.
11. McLean AHC, Parker BJ, Hrček J, Kavanagh JC, Wellham PAD, Godfray HCJ. Consequences of symbiont co-infections for insect host phenotypes. *J Anim Ecol* 2017; 1–11.
12. Weldon SR, Russell JA, Oliver KM. More is not always better: coinfections with defensive symbionts generate highly variable outcomes. *Appl Environ Microbiol* 2020; **86**.
